# The influence of multivitamins on neurological and growth disorders: a cross-sectional study

**DOI:** 10.3389/fnut.2024.1465875

**Published:** 2024-09-25

**Authors:** Jiaxiao Zhu, Penghong Xu, Wu Yan, Yahui Hu, Hongli Guo, Feng Chen, Francis Manyori Bigambo, Xu Wang

**Affiliations:** ^1^Department of Pediatrics, Nanjing Medical University, Nanjing, China; ^2^Department of Emergency, Pediatric Intensive Care Unit, Children’s Hospital of Nanjing Medical University, Nanjing, China; ^3^Clinical Research Center, Children's Hospital of Nanjing Medical University, Nanjing, China; ^4^Pharmaceutical Sciences Research Center, Department of Pharmacy, Children's Hospital of Nanjing Medical University, Nanjing, China

**Keywords:** 25(OH)D, vitamin A, vitamin B1, vitamin B2, vitamin B6, vitamin B12, multivitamins, vitamin E

## Abstract

**Background:**

While vitamin deficiencies can pose serious health consequences for the body, excessive intake of vitamins can also lead to health risks. However, there is limited data about the impact of multivitamins on neurological and growth disorders. This study aimed to investigate the relationship between multivitamins and neurological and growth disorders.

**Methods:**

A cross-sectional study was conducted with 16,921 subjects who visited the Children’s Hospital of Nanjing Medical University from 2019 to 2021. The subjects were categorized into two groups based on their health status including 9,368 cases (4,484 with neurological disorders and 4,884 with growth disorders) and 7,553 healthy controls. Statistical tests including the T-test, Wilcoxon Rank Sum test, and Chi-Square test were employed to compare the groups, and logistic regression and Weighted Quantile Sum (WQS) regression were used to identify associations.

**Results:**

In the adjusted logistic regression, serum 25 hydroxyvitamin D [25(OH)D], vitamin B2, and vitamin B9 were associated with decreasing risks of neurological disorders, whereas vitamin A, vitamin B1, and vitamin B12 were associated with increasing risks of neurological disorders. Nevertheless, vitamin A and vitamin B2 were associated with increasing risks of growth disorders. In the WQS model, nine multivitamins were positively associated with risks of neurological disorders, and Vitamins D and C were weighted the most. In addition, the inverse association but not statistically significant was observed between multivitamins and growth disorders, particularly growth retardation revealed a negative association, and some individual growth disorders revealed positive associations including obesity and malnutrition.

**Conclusion:**

In general, the study observed that multivitamins may be associated with neurological and growth disorders either positive or negative depending on the type of disorder.

## 1 Introduction

Vitamins are crucial for maintaining overall health and their deficiencies can pose serious health consequences, but excessive vitamin intake can also lead to health risks. Recent evidence strongly indicates that vitamins play a pivotal role in the initial brain development (1) and the growth and development of children (2). The combinations of nutrients may impact cancer risk more than individual nutrients (3). Proper multivitamin supplements are important for cardiovascular health (4), neural cognitive development (5), and physical growth. The developing world is plagued by a high frequency of multiple micro-nutrient deficiencies, notably iron, vitamin A, zinc, and iodine. In fact, vitamins play a vital role in maintaining the body’s balance, as any insufficiency can markedly disrupt normal metabolic functions (6). Inadequate support for neurological development in early life can have long-term effects on mental health and life quality (7, 8).

In the contemporary era, children are maturing under an unparalleled shift in food environments characterized by the persistence of nutritional challenges such as micro-nutrient deficiencies and food insecurity, alongside the escalating prevalence of overweight and obesity (9). Studies have found a link between prenatal or neonatal low 25 hydroxyvitamin D [25(OH)D] levels with neurological disorders such as autism (10, 11), attention deficit hyperactivity disorder (ADHD) (12), sleeping disorders, and headache and pain regulation (13). This insight has brought attention to the significance of 25(OH)D supplementation and the necessity of adequate plasma levels to prevent or relieve certain neurological disorders (13). Current observational studies consistently have demonstrated a connection between Vitamin B2 status or dietary intake and cognitive outcomes in children. It cannot be emphasized enough the significance of maintaining adequate levels of Vitamin B2, especially during pregnancy and early stage of childhood, given its crucial involvement in neural myelination, brain development, and the growth of fetuses and young children (14). Another study highlighted the connections between Vitamin A levels and the health of the visual system and epithelial tissue (15). Moreover, Vitamin A, and its derivative, retinoic acid, play pivotal roles in supporting regular metabolic functions, enhancing resistance against infections, and bolstering overall immunity (15). Notably, recent research has shown the significant roles of Vitamin A in mitigating the onset of metabolic disorders (16), influencing adipogenesis, lipid metabolism, and releasing bioactive substances. The shortage of Vitamin A not only hinders growth and development but also impacts various organ systems, including gastrointestinal, renal, and musculoskeletal systems (17).

Several previous studies have investigated the effects of individual vitamins on diseases, but the importance of multivitamins cannot be overlooked (18). Multivitamins offer significant benefits to the human body, not only due to the effects of individual vitamins but also because of the synergistic effects of multivitamins. Understanding the relationship between multivitamins with neurological and growth disorders is essential because it can assist in determining the optimal timing and mode of intervention and potentially averting any adverse effects of multivitamins. It has been reported that multivitamins have close relationships with various major systems of the human body and promote growth and development (19), but excessive multivitamin intake could also pose health risks (20). A study by Zhou et al. (5) is one of a few studies that addressed the combined effect of Vitamins A and B to treat attention-deficit/hyperactivity disorder (ADHD) in children. Tang et al. (21) also found a positive relationship between the mixture of vitamin A, vitamin B1, vitamin B2, vitamin B12, and vitamin D with obesity in children. Considering insufficient evidence for a relationship between multivitamins and diseases, particularly neurological and growth disorders in children, establishing practical nutritional guidelines poses a challenge. This underscores the necessity for further methodologically robust research to comprehensively explore the effects of multivitamins on various diseases (22). Our study is the first to specifically examine the impact of multivitamins on neurological and growth disorders in children via a large sample with multivitamin status measured in different diseases.

## 2 Materials and methods

### 2.1 Study design and participants

This cross-sectional study involved a total of 16,921 subjects who visited the Children’s Hospital of Nanjing Medical University between 2019 and 2021. The subjects were categorized into two groups based on their health status including 9,368 cases (4,484 with neurological disorders and 4,884 with growth disorders) and 7,553 healthy controls. Criteria for inclusion for the cases group were children who were diagnosed to have neurological disorders such as ADHD, anorexia, language development disorders, sleep disorders, Tourette syndrome, and/or growth disorders such as growth retardation, obesity, malnutrition, or breast development by experienced physicians. The Diagnostic and Statistical Manual of Mental Disorders, fourth (DSM-4) was used as the diagnostic criteria for neurological disorders. Growth disorders diagnostic criteria; Growth retardation was diagnosed when children’s heights were more than two standard deviations below the mean height for age and sex (23). Obesity was considered when BMI was ≥95th percentile for age (24). Malnutrition was defined when children’s Z-scores for height-for-age, weight-for-height, and weight-for-age measurements were below-2 standard deviations of the World Health Organization Child Growth Standards median (25). Breast development disorders were diagnosed using Tanner’s staging by a Chinese expert consensus (26, 27). The controls consisted of healthy children with neither neurological nor growth disorders. Our study adhered to the relevant STROBE guidelines. The Institutional Review Board of Children’s Hospital Nanjing Medical University approved the study, and the participants’ guardians provided informed consent. The information gathered was anonymous, meaning that it cannot show participants’ identifiable information.

### 2.2 Data collection methods

The participants’ comprehensive information was meticulously extracted from medical records, including sex, age, diagnosis, and a comprehensive set of vitamin levels. Age and sex age and gender were selected because are the most important characteristics of demography (28), and play a crucial role in determining the micronutrient needs of healthy individuals (29). The vitamin types detected include Vitamin D, specifically its 25-hydroxy form known as [25(OH)D] was known as a key indicator of Vitamin D status in the body; Vitamin A, essential for vision and immune function; and various B Vitamin including Vitamin B1 for energy metabolism, Vitamin B2 for cellular respiration, Vitamin B6 for protein metabolism, Vitamin B9 for DNA synthesis, and Vitamin B12 for nerve function and red blood cell formation. Moreover, the participants’ levels of Vitamin C, vital for immune support and collagen production, and Vitamin E, an antioxidant that helps protect cells from damage, were also included. To ensure accurate measurements, blood specimens were collected from each participant in the morning according to the sample collection protocol for to detection of serum vitamin levels. Serum vitamin levels were meticulously assayed using advanced high-performance liquid chromatography-triple quadrupole tandem mass spectrometry (LCMS-8050 CL, Shimadzu Corporation, Japan). The selection of vitamins was not only based on their relevance to the outcomes being studied but also on their public health implications.

### 2.3 Statistical analysis

We conducted this study via variables measured within a case–control study. Baseline characteristics were compared between both case and control groups using IBM SPSS (version 23). Descriptive statistics were expressed in mean ± standard deviation (SD) for continuous variables following normal distributions and an independent t-test was applied, whereas frequency and percentage (%) were expressed for categorical variables and the chi-square test was applied. For non-parametric data, especially vitamin concentrations, we calculated the median, 25th, and 75th percentiles for case and control groups using the Wilcoxon rank sum test according to the quartile of vitamin levels. To explore the association between vitamins and neurological and growth disorders, we used the multivariable logistic regression model to assess the individual effects of vitamins, the results were expressed as regression coefficient (OR) and 95% confidence intervals (CI) and Weighted Quantile Sum (WQS) regression model was utilized for multivitamins effects using R packages “gWQS.” Briefly, the WQS model creates a WQS index that estimates the overall effect of the mixture on the outcome of interest. The score is computed as a weighted sum of all exposures categorized into quartiles or quantiles to minimize the impact of outliers on weight estimation (30). In the current study, the WQS index and the weight of different vitamins were calculated to evaluate the joint effects of multivitamins on neurological and growth disorders. Model 1 was unadjusted and model 2 was further adjusted for age and gender. Bootstrap was 100, 60% of the samples were taken as the validation set to rigorously evaluate the model’s performance and minimize the risk of overfitting, 40% as the training set, and the seed was 2016 (30). All analysis was performed using R (version 4.2.1). A *p*-value of <0.05 was considered statistically significant.

## 3 Results

### 3.1 Basic characteristics of the study participants

**Figure 1:**
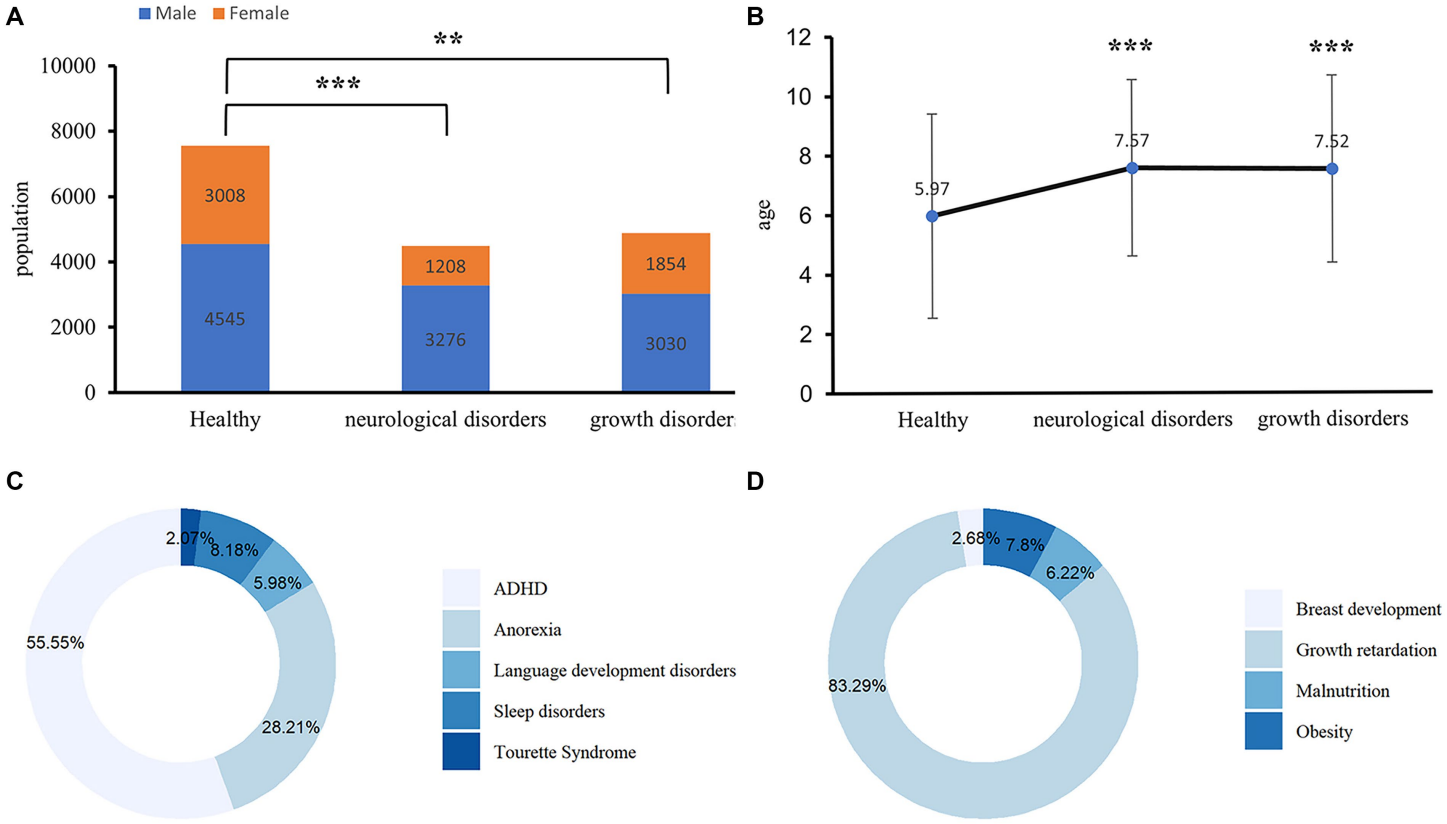
Baseline characteristics of children with neurological and growth disorders. **(A)** Gender difference between the cases and healthy controls among the subjects. The Chi-Square Test was applied to check for the difference, **p < 0.001, ***p < 0.0001. **(B)** Age difference between the cases and healthy controls, an independent *t*-test was applied to check for the difference, **p < 0.001, ***p < 0.0001. **(C)** The pie chart shows the types of diseases contained in neurological disorders. ADHD, attention-deficit/hyperactivity disorder. **(D)** The pie chart shows the types of diseases contained in growth disorders.

Figure 1 presents the basic characteristics of the study participants. Of the 16,921 participants enrolled in our study, 9,368 participants were divided into cases group with neurological disorders (*n* = 4,484) and growth disorders (*n* = 4,484), specifically, neurological disorders include ADHD (*n* = 2,491), anorexia (*n* = 1,265), language development disorders (*n* = 268), sleep disorders (*n* = 367), or Tourette syndrome (*n* = 93) and growth disorders include growth retardation (*n* = 4,068), obesity (*n* = 381), malnutrition (*n* = 304), or breast development (*n* = 131). The rest of 75,553 participants were healthy children in the control group. The mean age of the participants with neurological disorders was 7.57 years, and 3,276 (73.10%) were males; whereas the mean age of the participants with growth disorders was 7.52 years, and 3,030 (62.00%) were males. In the control group, the mean age of the healthy group was 5.97 years, and 4,545 (60.17%) were males. Age and gender were significant differences between participants with neurological disorders and the healthy control. Among the cases, the highest prevalent neurological disorder was ADHD (55.55%), followed by anorexia (28.21%), whereas, in growth disorders, growth retardation (83.29%) had the highest prevalence, followed by obesity (7.8%). Among neurological disorders, age and gender were significant differences in ADHD, language development disorders, sleep disorders, and Tourette syndrome compared with the healthy controls (Supplementary Table S1). In growth disorders, age and gender were significant differences in growth retardation, obesity, and breast development compared with the health controls, but in malnutrition, a significant difference was observed in age (Supplementary Table S2).

### 3.2 Laboratory characteristics of the study participants

Table 1 shows laboratory characteristics of children with neurological disorders (cases) and healthy groups (controls). Serum 25(OH)D (70.787 (52.776–95.464) vs. 75.330 (56.890–101.085) nmol/L, *p* < 0.001), Vitamin B1 (46.618 (31.871–60.468) vs. 48.884 (34.516–62.727) nmolL, *p* < 0.001), and Vitamin B2 (240.370 (54.606–333.223) vs. 253.113 (164.956–341.044) μg/L, *p* < 0.001) were significantly lower in children with neurological disorders compared with healthy control. Serum Vitamin A (0.787 (0.555–25.997) vs. 0.742 (0.534–14.447) μmol/L, *p* < 0.001), and serum Vitamin E (11.211 (10.807–12.243) vs. 11.141 (10.734–11.933) μmol/L, *p* < 0.001) were significantly higher in children with neurological disorders compared with healthy control. Among neurological disorders, serum 25(OH)D was significantly lower in ADHD and Tourette syndrome but was substantially higher in language development disorders and sleep disorders compared with the healthy controls. Serum vitamin A, vitamin B6, and vitamin B9 were significantly lower in anorexia, language development disorders, and sleep disorders, but serum vitamin A and vitamin B9 were substantially higher in ADHD compared with the healthy controls. Serum VB12 and VB2 were significantly higher in anorexia, language development disorders, and sleep disorders, but were significantly lower in ADHD compared with the healthy controls. On the other hand, VE was substantially lower in language development disorders and sleep disorders but was considerably higher in ADHD compared with the healthy controls. Serum vitamin C was significantly lower in ADHD compared with the healthy controls (Supplementary Table S3).

**Table 1:** Laboratory characteristics of children with neurological disorders and healthy controls.

Median (IQR)				
	Normal range	Cases (*N* = 4,484)	Healthy (*N* = 7,553)	*P*
25(OH)D	75--250 nmol/L	70.787 (52.776–95.464)	75.330 (56.890–101.085)	**<0.001**
VA	0.52--2.2 μmol/L	0.787 (0.555–25.997)	0.742 (0.534–14.447)	**<0.001**
VB1	50--150nmolL	46.618 (31.871–60.468)	48.884 (34.516–62.727)	**<0.001**
VB12	200--900 pg./L	261.769 (31.252–357.160)	269.573 (84.550–359.680)	**0.002**
VB2	>200 μg/L	240.370 (54.606–333.223)	253.113 (164.956–341.044)	**<0.001**
VB6	14.6--72.9 μmol/L	30.752 (22.884–38.927)	30.457 (22.401–38.931)	0.111
VB9	6.8--36.3 nmol/L	11.405 (8.823–30.442)	11.475 (8.970–17.844)	0.864
VC	34--114 μmol/L	34.020 (29.656–39.454)	34.303 (30.420–39.785)	**0.024**
VE	10--15 μg/mL	11.211 (10.807–12.243)	11.141 (10.734–11.933)	**<0.001**

25(OH)D, 25-hydroxyvitamin D; VA, vitamin A; VB1, vitamin B1; VB2, vitamin B2; VB6, vitamin B6; VB9, vitamin B9; VB12, vitamin B12; VC, vitamin C; VE, vitamin E; *p* < 0.05 was considered statistically significant. The bold values represent statistically significant results.

Table 2 presents laboratory characteristics of children with growth disorders and healthy controls. Serum 25(OH)D (67.885 (48.538–92.908) vs. 75.330 (56.890–101.085) nmol/L, (*p* < 0.001), serum Vitamin B2 (239.380 (50.095–334.802) vs. 253.113 (164.956–341.044) μg/L, *p* < 0.001), and serum Vitamin C (33.856 (28.813–39.291) vs. 34.303 (30.420–39.785) μmol/L, p < 0.001) were significantly lower in children with growth disorders compared to healthy control. On the other hand, serum Vitamin A (0.809 (0.557–35.141) vs. 0.742 (0.534–14.447) μmol/L, *p* < 0.001), and serum Vitamin E (11.277 (10.816–12.709) vs. 11.141 (10.734–11.933)) μmol/L, *p* < 0.001) were significantly higher in children with growth disorders compared with healthy control. Among growth disorders, serum 25(OH)D was significantly lower in growth retardation, obesity, and breast development compared with the healthy controls. In contrast, serum vitamin A was substantially higher in growth retardation, obesity, and malnutrition compared with the healthy controls. Serum vitamin C was significantly higher in obesity and lower in growth retardation compared with the healthy controls. On the other hand, serum vitamin B1, vitamin B12, and vitamin B2 were significantly lower in growth retardation and malnutrition compared with the healthy controls. In contrast, serum vitamin E was substantially higher in growth retardation (Supplementary Table S4).

**Table 2:** Laboratory characteristics of children with growth disorders and healthy controls.

Median (IQR)				
	Normal range	Cases (*N* = 4,884)	Healthy (*N* = 7,553)	*P*
25(OH)D	75--250 nmol/L	67.885 (48.538–92.908)	75.330 (56.890–101.085)	**<0.001**
VA	0.52--2.2 μmol/L	0.809 (0.557–35.141)	0.742 (0.534–14.447)	**<0.001**
VB1	50--150nmolL	48.247 (34.258–63.536)	48.884 (34.516–62.727)	0.881
VB12	200--900 pg./L	259.646 (38.147–355.107)	269.573 (84.550–359.680)	**0.012**
VB2	>200 μg/L	239.380 (50.095–334.802)	253.113 (164.956–341.044)	**<0.001**
VB6	14.6--72.9 μmol/L	30.018 (20.821–39.482)	30.457 (22.401–38.931)	**0.045**
VB9	6.8--36.3 nmol/L	11.249 (8.856–20.131)	11.475 (8.970–17.844)	0.280
VC	34--114 μmol/L	33.856 (28.813–39.291)	34.303 (30.420–39.785)	**<0.001**
VE	10--15 μg/mL	11.277 (10.816–12.709)	11.141 (10.734–11.933)	**<0.001**

25(OH)D, 25-hydroxyvitamin D; VA, vitamin A; VB1, vitamin B1; VB2, vitamin B2; VB6, vitamin B6; VB9, vitamin B9; VB12, vitamin B12; VC, vitamin C; VE, vitamin E; *P* < 0.05 was considered statistically significant. The bold values represent statistically significant results.

### 3.3 Logistic regression analysis to examine the association between individual vitamins and neurological and growth disorders

We performed logistic regression to evaluate the association of vitamins with neurological and growth disorders. The adjusted logistic regression results showed that serum 25(OH)D (OR = 0.998, 95% CI: 0.997 ~ 0.998; *p* < 0.001), vitamin B2 (OR = 0.999, 95% CI: 0.999 ~ 1.000; *p* < 0.001), and vitamin B9 (OR = 0.999, 95% CI: 0.998 ~ 1.000; *p* < 0.001) were associated with decreasing risks of neurological disorders, whereas vitamin A (OR = 1.003, 95% CI: 1.002 ~ 1.004; *p* < 0.001), vitamin B1 (OR = 1.001, 95%CI:1.000 ~ 1.002; *p* = 0.014), and vitamin B12 (OR = 1.000, 95%CI: 0.999 ~ 1.000, *p* = 0.023) were associated with increased risks of neurological disorders. On the other hand, vitamin A (OR = 1.001, 95% CI: 1.001 ~ 1.002; *p* = 0.001) and vitamin B2 (OR = 1.000, 95%CI: 0.999 ~ 1.000; *p* = 0.027) were associated with increasing risks of growth disorders (Table 3).

**Table 3:** Associations between vitamin levels with neurological and growth disorders.

	Neurological disorders				Growth disorders			
	Model 1 OR (95%CI)	*P*	Model 2 OR (95%CI)	*P*	Model 1 OR (95% CI)	*P*	Model 2 OR (95% CI)	*P*
25(OH)D	0.999 (0.998 ~ 0.999)	<0.001	0.998 (0.997 ~ 0.998)	**<0.001**	1.000 (1.000 ~ 1.001)	0.078	1.000 (0.999 ~ 1.000)	0.052
VA	1.002 (1.001 ~ 1.003)	<0.001	1.003 (1.002 ~ 1.004)	**<0.001**	1.001 (1.000 ~ 1.002)	0.037	1.001 (1.001 ~ 1.002)	**0.001**
VB1	1.001 (1.000 ~ 1.002)	0.027	1.001 (1.000 ~ 1.002)	**0.014**	0.999 (0.998 ~ 1.001)	0.357	0.999 (0.998 ~ 1.000)	0.108
VB12	1.000 (1.000 ~ 1.000)	0.562	1.000 (0.999 ~ 1.000)	**0.023**	1.000 (1.000 ~ 1.000)	0.266	1.000 (1.000 ~ 1.000)	0.387
VB2	1.000 (0.999 ~ 1.000)	0.019	0.999 (0.999 ~ 1.000)	**<0.001**	1.000 (1.000 ~ 1.000)	0.598	1.000 (0.999 ~ 1.000)	**0.027**
VB6	1.000 (0.999 ~ 1.000)	0.232	1.001 (1.000 ~ 1.001)	0.077	0.999 (0.999 ~ 1.000)	0.100	1.000 (1.000 ~ 1.001)	0.223
VB9	0.999 (0.998 ~ 1.000)	0.005	0.999 (0.998 ~ 1.000)	**0.005**	0.999 (0.999 ~ 1.000)	0.049	0.999 (0.999 ~ 1.000)	0.058
VC	1.000 (0.999 ~ 1.001)	0.887	1.000 (0.999 ~ 1.000)	0.348	1.001 (1.000 ~ 1.002)	0.031	1.001 (1.000 ~ 1.001)	0.131
VE	1.001 (1.000 ~ 1.001)	0.107	1.000 (1.000 ~ 1.001)	0.281	1.000 (0.999 ~ 1.001)	0.856	1.000 (0.999 ~ 1.000)	0.527

25(OH)D, 25-hydroxyvitamin D; VA, vitamin A; VB1, vitamin B1; VB2, vitamin B2; VB6, vitamin B6; VB9, vitamin B9; VB12, vitamin B12; VC, vitamin C; VE, vitamin E; *P* < 0.05 was considered statistically significant. Model 1 was unadjusted; Model 2 was adjusted for child’s age and gender. The bold values represent statistically significant results.

### 3.4 Weighted quantile sum regression analysis to examine the combined association between multivitamins and neurological and growth disorders

Table 4 shows associations between the WQS regression indexes of multivitamins with neurological and growth disorders. In the unadjusted model, nine multivitamins revealed significant positive correlations with neurological disorders (OR = 1.060, 95% CI: 1.043–1.078; *p* < 0.001), particularly, ADHD (OR = 1.115, 95% CI: 1.100–1.130; *p* < 0.001), sleep disorders (OR = 1.028, 95% CI: 1.017–1.039; *p* < 0.001), anorexia (OR = 1.032, 95% CI: 1.021–1.043; *p* < 0.001), and language development disorders (OR = 1.020, 95% CI: 1.012–1.029; *p* < 0.001) as well as growth disorders (OR = 1.029, 95% CI: 1.016–1.042; *p* < 0.001), including obesity (OR = 1.017, 95% CI: 1.006–1.028; *p* = 0.002) and malnutrition (OR = 1.012, 95% CI: 1.004–1.021; *p* = 0.003). However, in the adjusted model, multivitamins were consistently positively associated with neurological disorders (OR = 1.029, 95% CI: 1.009–1.050; *p* = 0.005), particularly ADHD (OR = 1.064, 95% CI: 1.049–1.078; *p* < 0.001), while other neurological disorders including anorexia (OR = 0.954, 95% CI: 0.942–0.966; *p* < 0.001) and language development disorders (OR = 0.967, 95% CI: 0.958–0.976; *p* < 0.001) revealed negative associations. Nevertheless, the inverse association but not statistically significant was observed between multivitamins and growth disorders (OR = 0.993, 95% CI: 0.976–1.010; *p* < 0.398), particularly growth retardation (OR = 0.968, 95% CI: 0.949–0.989; *p* = 0.002), and some individuals growth disorders revealed positive associations including obesity (OR = 1.012, 95% CI: 1.003–1.021; *p* = 0.010) and malnutrition (OR = 1.011, 95% CI: 1.001–1.021; *p* = 0.031).

**Table 4:** Associations between the weighted quantile sum regression indexes of multivitamins with neurological and growth disorders.

	Model 1		Model 2	
	OR_WQS_ (95%CI)	*P*	OR_WQS_ (95%CI)	*P*
Neurological disorders	1.060 (1.043–1.078)	**<0.001**	1.029 (1.009–1.050)	**0.005**
ADHD	1.115 (1.100–1.130)	**<0.001**	1.064 (1.049–1.078)	**<0.001**
Tourette syndrome	0.999 (0.995–1.003)	0.571	0.998 (0.992–1.005)	0.609
Sleep disorders	1.028 (1.017–1.039)	**<0.001**	1.012 (0.999–1.025)	0.074
Anorexia	1.032 (1.021–1.043)	**<0.001**	0.954 (0.942–0.966)	**<0.001**
Language development disorders	1.020 (1.012–1.029)	**<0.001**	0.967 (0.958–0.976)	**<0.001**
Growth disorders	1.029 (1.016–1.042)	**<0.001**	0.993 (0.976–1.010)	0.398
Growth retardation	0.930 (0.910–0.951)	**<0.001**	0.968 (0.949–0.989)	**0.002**
Obesity	1.017 (1.006–1.028)	**0.002**	1.012 (1.003–1.021)	**0.010**
Breast development	0.999 (0.990–1.008)	0.836	1.001 (0.993–1.010)	0.745
Malnutrition	1.012 (1.004–1.021)	**0.003**	1.011 (1.001–1.021)	**0.031**

Model 1 was the unadjusted model; Model 2 was adjusted for child’s age and gender. The bold values represent statistically significant results.

**Figure 2:**
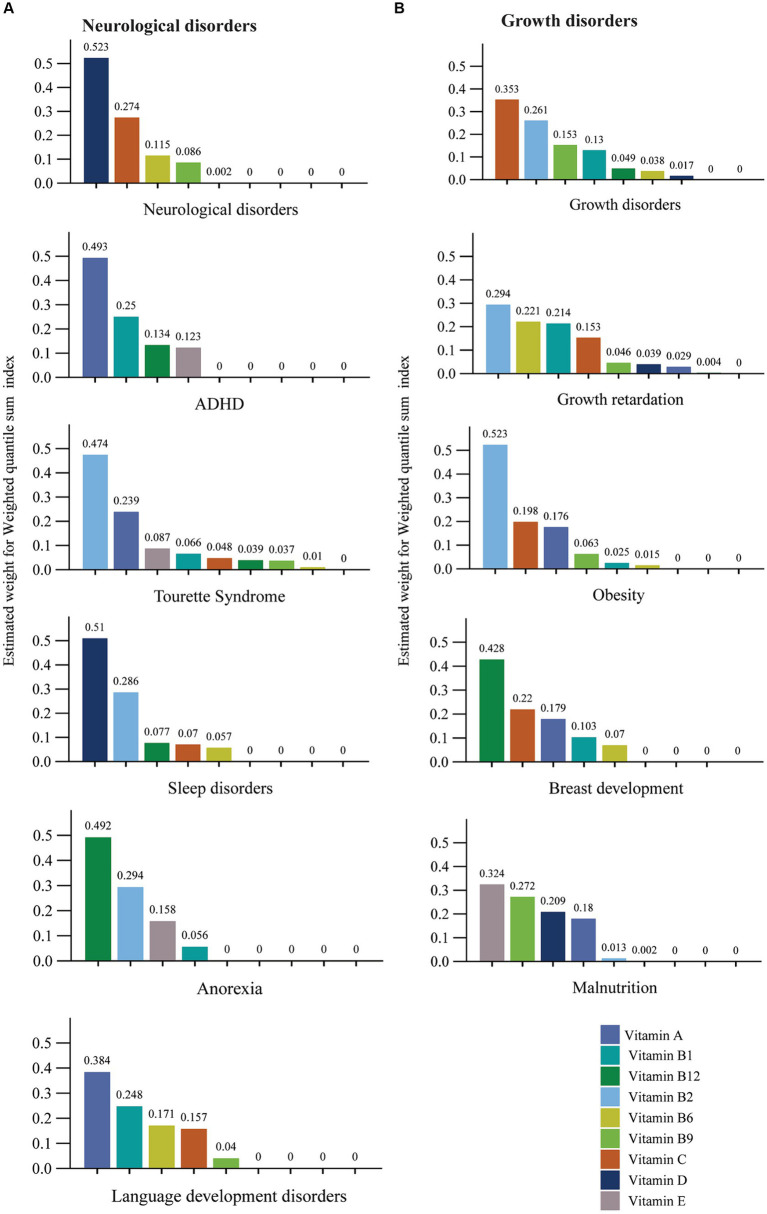
The estimated weight for each weighted quantile sum (WQS) index of multivitamins associated with different type of diseases. **(A)** Neurological disorders. **(B)** Growth disorders. Models were adjusted for gender and age.

Figure 2 estimates the weight for each WQS index of multivitamins associated with different types of diseases. The adjusted WQS regression results showed that Vitamin D and C, respectively, weigh the highest neurological disorders and growth disorders. The WQS regression model also suggests that the weights of different vitamins vary in different diseases, for instance, Vitamin B2 weighs the most in the Tourette syndrome, growth retardation, and obesity. Vitamin D had the highest weight among multivitamins in the WQS index related to sleep disorders. Vitamin A had the top weight in the WQS index for ADHD and language development disorders. Moreover, for anorexia and breast development, Vitamin B12 prevails over other multivitamins in the WQS index. Vitamin E ranks highest in the WQS index for malnutrition.

## 4 Discussion

### 4.1 Principal findings

This research assessed associations between multivitamins with neurological and growth disorders using logistic regression and the WQS regression. In the adjusted logistic regression, serum 25(OH)D, vitamin B2, and vitamin B9 were associated with decreasing risks of neurological disorders, whereas vitamin A, vitamin B1, and vitamin B12 were associated with increasing risks of neurological disorders. Nevertheless, vitamin A and vitamin B2 were associated with increasing risks of growth disorders. In the adjusted WQS model, we found that multivitamins were positively associated with neurological disorders, particularly, ADHD, while other neurological disorders including anorexia and language development disorders revealed negative associations. On the other hand, the inverse association but not statistically significant was observed between multivitamins and growth disorders, particularly growth retardation revealed a negative association, and some individual growth disorders revealed positive associations including obesity and malnutrition.

### 4.2 Multivitamins and neurological and growth disorders

Multivitamins offer significant benefits to the human body, not only due to the effects of individual vitamins but also because of the synergistic effects of multivitamins. Understanding the relationship between multivitamins with neurological and growth disorders is essential because it can assist in determining the optimal timing and mode of intervention and potentially averting any adverse effects of multivitamins. It has been reported that multivitamins have close relationships with various major systems of the human body and promote growth and development (19), but excessive multivitamin intake could also pose health risks (20). Epidemiological studies have reported contradictory results about the benefits of multivitamins in preventing chronic disease. For instance, a study involving one million U. S adults found a decrease in the risk of cardiovascular mortality among users of multivitamin/multimineral supplements (MVMS) as well as vitamin A, vitamin C, and/or vitamin E. The same study further found an increase in the cancer mortality risk in male MVMS users who smoked (31). Two epidemiological studies have shown a decrease in nonfatal myocardial infarction in the subjects on regular and occasional dietary supplements such as single nutrients and MVMS compared to those not on dietary supplements. In contrast, other studies did not find a relationship between multivitamins with the risk of cancer (32, 33) and cardiovascular diseases (33). Our findings were somewhat similar to the previous findings as we found that multivitamins were associated with increasing risks of ADHD, obesity, and malnutrition. On the other hand, multivitamins were related to decreased risks of growth retardation, anorexia, and language development disorders. The underlying mechanisms for these relationships are complex and may be influenced by numerous factors. The possible explanation for these relationships could be that children may profoundly rely on multivitamins and ignore a balanced diet. This can lead to insufficient intake of nutrients, leading to malnutrition or obesity despite taking supplements. On the other hand, vitamin B2 and vitamin B12 play a key role in one-carbon metabolism and the formation of methyl donors. The co-presence of vitamin B2 and vitamin B12 in multivitamins could lead to an increased risk of obesity (21). Vitamins act as cofactors in the brain for various biochemical pathways and play key roles in neurotransmitter formation such as serotonin and dopamine, which participate in ADHD-related symptoms (34). We hypothesized that multivitamins may alter the level of neurotransmitters such as dopamine and serotonin, consequently increasing the risk of ADHD. Moreover, multivitamins can provide essential nutrients that are essential for growth and cognitive development (7, 35), thereby reducing risks of developmental delay such as growth retardation and language delay. Because of the scarcity of data, further studies are needed to substantiate this hypothesis.

### 4.3 Individual vitamins and neurological and growth disorders

We assessed the associations between individual vitamins with neurological and growth disorders, in logistic regression, we found that 25 (OH)D and Vitamin B2 were associated with decreasing risks of neurological disorders, while vitamin A was associated with increasing risks of both neurological and growth disorders. In addition, we observed significantly lower serum 25(OH)D and vitamin B2 in children with neurological disorders compared to healthy controls, while vitamin A and vitamin E were both significantly higher in children with neurological and growth disorders compared to healthy controls.

Despite neurological and growth disorders having complex mechanisms that depend on genetic, environmental, nutritional, and hormonal factors (36), our findings suggest a potential association of these vitamins with neurological and growth disorders. For instance, we observed a significantly lower 25(OH)D among children with neurological disorders such as ADHD and Tourette syndrome as well as growth disorders like growth retardation, obesity, and breast development compared to healthy control. Research evidence suggests that 25(OH)D supports normal brain growth, and increases neural protection (37), and anti-inflammatory mechanisms, which are vital components of the brain. Indeed, low serum 25(OH)D levels at birth have been linked to an increased risk of persistent short sleepers among children aged 2 to 6 years (38). Conversely, existing literature demonstrates a positive association between cognitive function and 25(OH)D status (39). This suggests that 25(OH)D potentially exhibits neuron protective functions by mitigating glutamate-induced neurotoxin (40) and modulating the genetic expression of diverse proteins. As one of the hormones, 25(OH)D intricately regulates gene expression and operates within multiple signaling pathways, exerting intricate effects on adipocytes. Beyond its neurological implications, 25(OH)D significantly influences other physiological systems, including the cardiovascular and endocrine systems (41).

Obesity belongs to growth disorder, and it is closely correlated with insufficient levels of 25(OH)D. However, despite efforts at weight loss, the relationship between weight reduction and improved 25(OH)D levels appears to have minimal impact (42). Moreover, studies indicate that the supplementation of 25(OH)D would not lead to weight loss (43), suggesting that the use of 25(OH)D should not be considered as a method for weight reduction. This association could potentially be explained by the significant impact of obesity on 25(OH)D status and metabolism. Notably, obese children and adolescents would be particularly susceptible to 25(OH)D insufficiency or deficiency (44).

Vitamin B2, also known as Riboflavin, is a water-soluble compound recognized for its role as a free radical scavenger and contains antioxidant properties in the growth and reproduction processes of both humans and animals (45). Its biochemical function lies in serving as a precursor for essential co-enzymes, namely Flavin Adenine Dinucleotide (FAD) and Flavin Mononucleotide (FMN), thereby playing a pivotal role in facilitating oxidation–reduction reactions across all living organisms (46). In our study, serum Vitamin B2 was associated with a decreasing risk of neurological disorders but was also associated with an increasing risk of growth disorders in the logistic regression. However, the serum Vitamin B2 was significantly lower in children with both neurological and growth disorders compared to the healthy controls. Meanwhile, research evidence suggests that riboflavin has multiple effects on several cellular pathways, some of them relevant to mechanisms of neuron degeneration, shared by the main neurological diseases (47).

Vitamin C is a crucial antioxidant in the brain and has been reported to have numerous functions, including neuron modulation and involvement in angiogenesis. The absence of Vitamin C in the brain is detrimental to cognitive decline (48). In our study, Vitamin C was neither associated with neurological disorders nor growth disorders. However, it was observed to be lower in children with both neurological and growth disorders. Similarly, in animal tests, Vitamin C increased the fecal fat excretion by chitosan in guinea pigs, thereby reducing body weight gain (49).

Vitamins A and E are important fat-soluble micro-nutrients that play a great role in metabolism, growth, and development as well as maintaining normal physiological activities of the body (50). Another study has reported Vitamin E to be a protective agent for neuron degeneration, cardiovascular, and cancer through its antioxidant features or other molecular mechanisms (51). In our study, Vitamin A and E were significantly higher in both children with neurological and growth disorders according to healthy children. However, the logistic regression results showed that Vitamin A was statistically significantly associated with increasing risk of both neurological and growth disorders, but Vitamin E revealed no significant associations. On the contrary, previous studies have observed lower levels of vitamin A in children with neurological disorders compared with healthy children, particularly autism spectrum disorder (52, 53). Another study has found higher serum Vitamins A and E to be associated with neuron-generative diseases, specifically amyotrophic lateral sclerosis (54), which may partly be similar to our findings that vitamin A might be associated with an increasing risk of neurological disorders. On the other hand, Vitamin A deficiency has been reported to be associated with growth disorders, particularly early childhood stunting (55). Other studies have found positive associations between Vitamin A with obesity, metabolic syndrome, hyperuricemia, and dyslipidemia in children and adolescents (56). The discrepancy observed between various studies could potentially be attributed to the diverse types of diseases that have been investigated. Each disease possesses its unique pathophysiology, etiology, and progression, which can significantly influence the outcomes of research findings. For instance, some diseases might respond favorably to certain interventions while others might not, and this would lead to conflicting results across studies. Therefore, it is crucial to take into account the specific nature of the diseases studied when comparing and analyzing research outcomes. In our study, we explored neurological disorders such as ADHD, anorexia, language development disorders, sleep disorders, and Tourette syndrome, as well as growth disorders such as growth retardation, obesity, malnutrition, and breast development. After all, the effectiveness of vitamins in treating or preventing a specific disease depends on a range of factors, including the type of disease, the individual’s health status, and the dosage and form of the vitamin being used. In addition, even though the levels of vitamins A and E were higher in children with neurological and growth disorders compared with the healthy controls, still were in the normal ranges. Therefore, it is essential to further investigate the potential benefits of vitamin E and vitamin A concentration in humans. A study has not recommended the use of vitamin supplements to reduce the risk of non-communicable diseases in the general population in the absence of clinical nutritional deficiency (57).

Overall, the physiological functions of vitamins are complex and not fully elucidated, and different vitamins have different effects on the occurrence and development of diseases, but one prospective study has introduced a bidirectional association of neuron development with growth (58). Since vitamin levels are involved in the processes of neurological and physical development, there might be a correlation between changes in vitamin levels with neurological and growth disorders. It is plausible that vitamins, particularly vitamin A, vitamin B, vitamin C, and vitamin E play significant roles in neurological and growth disorders through various mechanisms, including epigenetic reprogramming, the involvement of metabolic control, and inflammatory processes (21, 43, 59, 60).

### 4.4 Strength and limitations

The strengths of this study. This cross-sectional study provides new clues for studying the relationships between multivitamins and diseases. In particular, it emphasizes the importance of comprehensive and systematic thinking in understanding the complexity of relationships between multivitamins and neurological and growth disorders. In addition, we used weighted quantile sum regression to assess the overall effect of multivitamins on neurological and growth disorders.

Moreover, we recognize that there are several limitations in this study. Firstly, in our initial comparison of alterations in vitamin levels, we utilized the standard laboratory values for vitamins as stipulated by Chinese standards. It is essential to consider that diverse countries establish and employ their distinct recommended standards due to variations in food diversity and disparities in population demographics. Secondly, the scope of our findings was constrained by limited due to the cross-sectional survey design so causal relationships have not been established. Thirdly, a few co-variates were adjusted in the regression models which could lead to unmeasured confounding bias.

## 5 Conclusion

This study indicates that serum 25 (OH)D and vitamin B2 were associated with decreasing risks of neurological disorders, while vitamin A was associated with increasing risks of neurological and growth disorders. In addition, we observed significantly lower serum 25(OH)D and vitamin B2 in children with neurological disorders compared to healthy controls, while vitamin A and vitamin E were both significantly higher in children with neurological and growth disorders compared to healthy controls. Moreover, we observed that multivitamins may be differently associated with neurological and growth disorders either positive or negative depending on the type of disease. Consequently, there is a necessity for further investigations aimed at elucidating the precise mechanisms through which these parameters contribute to the etiology and potential treatments for neurological and growth disorders. Moreover, a prospective cohort study is required to validate the clinical implications of multivitamins on neurological and growth disorders.

## Data Availability

The raw data supporting the conclusions of this article will be made available by the authors, without undue reservation.
