# Supplementary material for: The influence of multivitamins on neurological and growth disorders: a cross-sectional study
Source: Front Nutr. 2024 Sep 25;11:1465875. doi: 10.3389/fnut.2024.1465875 (PMC11463060; doi:10.3389/fnut.2024.1465875)
Supplement: Supplementary file 1 [file Table_1.DOCX]

Supplementary Material

# Supplementary Tables

**Table S1. Baseline characteristics of various diseases in neurological disorders and healthy controls**

| Characteristics | | Cases | | | Healthy | *P* |
| --- | --- | --- | --- | --- | --- | --- |
| **ADHD** | |  | | |  |  |
|  | | N=2491 | | | N=7553 |  |
| Age(years) | | 9.12 ± 2.18 | | | 5.97 ± 3.44 | **<0.001** |
| Gender |  |  | | |  | **<0.001** |
| Male |  | 1993 (80.01) | | | 4545 (60.17) |  |
| Female |  | 498 (19.99) | | | 3008 (39.83) |  |
| **Anorexia** |  |  | | |  |  |
|  |  | N=1265 | | | N=7553 |  |
| Age(years) | | 6.11 ± 2.66 | | | 5.97 ± 3.44 | 0.113 |
| Gender |  |  | | |  | 0.113 |
| Male |  | 791 (62.53) | | | 4545 (60.17) |  |
| Female |  | 474 (37.47) | | | 3008 (39.83) |  |
| **Language Development Disorders** | | |  |  |  |  |
|  |  | N=268 | | | N=7553 |  |
| Age(years) |  | 4.22±0.80 | | | 5.97 ± 3.44 | **<0.001** |
| Gender |  |  | | |  | **<0.001** |
| Male |  | 213 (79.48) | | | 4545 (60.17) |  |
| Female |  | 55 (20.52) | | | 3008 (39.83) |  |
| **Sleep disorders** |  |  | | |  |  |
|  |  | N=367 | | | N=7553 |  |
| Age(years) |  | 4.22±0.80 | | | 5.97 ± 3.44 | **0.05** |
| Gender |  |  | | |  | **0.05** |
| Male |  | 202 (55.04) | | | 4545 (60.17) |  |
| Female |  | 165 (44.96) | | | 3008 (39.83) |  |
| **Tourette Syndrome** |  |  | | |  |  |
|  |  | N=93 | | | N=7553 |  |
| Age(years) |  | 8.48 ± 2.29 | | | 5.97 ± 3.44 | **<0.001** |
| Gender |  |  | | |  | **<0.001** |
| Male |  | 77 (82.80) | | | 4545 (60.17) |  |
| Female |  | 16 (17.20) | | | 3008 (39.83) |  |

25(OH)D, 25-hydroxyvitamin D; VA, vitamin A; VB1, vitamin B1; VB2, vitamin B2; VB6, vitamin B6; VB9, vitamin B9; VB12, vitamin B12; VC, vitamin C; VE, vitamin E; ADHD, attention-deficit/hyperactivity disorder. *P* <0.05 was considered statistically significant.

**Table S2. Baseline characteristics of various diseases in growth disorders and healthy controls**

| Characteristics | | Cases | Healthy | *P* |
| --- | --- | --- | --- | --- |
| **Growth retardation** | |  |  |  |
|  | | N=4068 | N=7553 |  |
| Age(years) | | 7.36 ± 3.09 | 5.97 ± 3.44 | **0.001** |
| Gender |  |  |  | **0.001** |
| Male |  | 2582 (63.47) | 4545 (60.17) |  |
| Female |  | 1486 (36.53) | 3008 (39.83) |  |
| **Obesity** |  |  |  |  |
|  |  | N=381 | N=7553 |  |
| Age(years) | | 9.69 ± 2.83 | 5.97 ± 3.44 | **<0.001** |
| Gender |  |  |  | **<0.001** |
| Male |  | 275 (72.18) | 4545 (60.17) |  |
| Female |  | 106 (27.82) | 3008 (39.83) |  |
| **Malnutrition** |  |  |  |  |
|  |  | N=304 | N=7553 |  |
| Age(years) | | 6.42 ± 3.49 | 5.97 ± 3.44 | **0.025** |
| Gender |  |  |  | 0.171 |
| Male |  | 171 (56.25) | 4545 (60.17) |  |
| Female |  | 133 (43.75) | 3008 (39.83) |  |
| **breast development** |  |  |  |  |
|  |  | N=131 | N=7553 |  |
| Age(years) | | 8.58 ± 2.02 | 5.97 ± 3.44 | **<0.001** |
| Gender |  |  |  | **<0.001** |
| Male |  | 2 (1.53) | 4545 (60.17) |  |
| Female |  | 129 (98.47) | 3008 (39.83) |  |

*P* <0.05 was considered statistically significant.

**Table S3. Laboratory characteristics of children in cases and healthy controls**

|  |  | Median (IQR) | | | | |  |
| --- | --- | --- | --- | --- | --- | --- | --- |
|  | Normal Range | Cases (Neurological disorders) | | | | Healthy | *P* |
| **ADHD**  25(OH)D | 75--250nmol/L | 60.055 (41.712-81.760) | | | | 75.330 (56.890-101.085) | **<0.001** |
| VA | 0.52--2.2umol/L | 13.741 (0.686-57.279) | | | | 0.742 (0.534-14.447) | **<0.001** |
| VB1 | 50--150nmolL | 47.518 (31.574-65.351) | | | | 48.884 (34.516-62.727) | 0.180 |
| VB12 | 200--900pg/L | 205.180 (17.217-324.773) | | | | 269.573 (84.550-359.680) | **<0.001** |
| VB2 | >200ug/L | 206.591 (31.642-311.757) | | | | 253.113 (164.956-341.044) | **<0.001** |
| VB6 | 14.6--72.9umol/L | 31.287 (19.609-43.421) | | | | 30.457 (22.401-38.931) | 0.157 |
| VB9 | 6.8--36.3nmol/L | 12.103 (9.481-36.636) | | | | 11.475 (8.970-17.844) | **<0.001** |
| VC | 34--114umol/L | 33.777 (12.150-41.892) | | | | 34.303 (30.420-39.785) | **<0.001** |
| VE | 10--15ug/mL | 11.571 (10.876-35.867) | | | | 11.141 (10.734-11.933) | **<0.001** |
| **Anorexia** |  |  | | | |  |  |
| 25(OH)D | 75--250nmol/L | 74.017 (57.109-95.102) | | | | 75.330 (56.890-101.085) | 0.055 |
| VA | 0.52--2.2umol/L | 0.648 (0.500-0.939) | | | | 0.742 (0.534-14.447) | **<0.001** |
| VB1 | 50--150nmolL | 50.039 (38.223-63.161) | | | | 48.884 (34.516-62.727) | **0.033** |
| VB12 | 200--900pg/L | 302.883 (224.338-376.920) | | | | 269.573 (84.550-359.680) | **<0.001** |
| VB2 | >200ug/L | 260.866 (210.006-352.336) | | | | 253.113 (164.956-341.044) | **<0.001** |
| VB6 | 14.6--72.9umol/L | 29.070 (21.671-36.276) | | | | 30.457 (22.401-38.931) | **<0.001** |
| VB9 | 6.8--36.3nmol/L | 10.572 (8.600-13.982) | | | | 11.475 (8.970-17.844) | **<0.001** |
| VC | 34--114umol/L | 33.953 (30.941-38.107) | | | | 34.303 (30.420-39.785) | 0.228 |
| VE | 10--15ug/mL | 11.137 (10.810-11.732) | | | | 11.141 (10.734-11.933) | 0.851 |
| **Language Development Disorders** | | |  |  | |  |  |
| 25(OH)D | 75--250nmol/L | 81.162 (65.781-97.349) | | | | 75.330 (56.890-101.085) | **0.025** |
| VA | 0.52--2.2umol/L | 0.586 (0.458-0.742) | | | | 0.742 (0.534-14.447) | **<0.001** |
| VB1 | 50--150nmolL | 46.846 (38.887-55.654) | | | | 48.884 (34.516-62.727) | 0.271 |
| VB12 | 200--900pg/L | 300.126 (233.169-380.620) | | | | 269.573 (84.550-359.680) | **<0.001** |
| VB2 | >200ug/L | 265.606 (219.901-370.498) | | | | 253.113 (164.956-341.044) | **<0.001** |
| VB6 | 14.6--72.9umol/L | 27.974 (21.261-34.863) | | | | 30.457 (22.401-38.931) | **0.001** |
| VB9 | 6.8--36.3nmol/L | 9.981 (7.962-12.420) | | | | 11.475 (8.970-17.844) | **<0.001** |
| VC | 34--114umol/L | 33.784 (31.115-36.718) | | | | 34.303 (30.420-39.785) | 0.088 |
| VE | 10--15ug/mL | 11.047 (10.782-11.350) | | | | 11.141 (10.734-11.933) | **0.002** |
| **Sleep disorders** | | | | | | | |
| 25(OH)D | 75--250nmol/L | 85.430 (67.220-110.050) | | | | 75.330 (56.890-101.085) | **<0.001** |
| VA | 0.52--2.2umol/L | 0.629 (0.513-0.850) | | | | 0.742 (0.534-14.447) | **<0.001** |
| VB1 | 50--150nmolL | 49.918 (37.237-62.965) | | | | 48.884 (34.516-62.727) | 0.193 |
| VB12 | 200--900pg/L | 306.004 (237.054-389.863) | | | | 269.573 (84.550-359.680) | **<0.001** |
| VB2 | >200ug/L | 270.840 (217.240-356.360) | | | | 253.113 (164.956-341.044) | **<0.001** |
| VB6 | 14.6--72.9umol/L | 29.398 (22.199-37.040) | | | | 30.457 (22.401-38.931) | **<0.001** |
| VB9 | 6.8--36.3nmol/L | 10.678 (8.609-13.307) | | | | 11.475 (8.970-17.844) | **<0.001** |
| VC | 34--114umol/L | 33.772 (31.017-37.945) | | | | 34.303 (30.420-39.785) | 0.436 |
| VE | 10--15ug/mL | 11.067 (10.709-11.518) | | | | 11.141 (10.734-11.933) | **0.002** |
| **Tourette Syndrome** | |  | | |  |  |  |
| 25(OH)D | 75--250nmol/L | 56.507 (44.880-73.819) | | | | 75.330 (56.890-101.085) | **<0.001** |
| VA | 0.52--2.2umol/L | 0.823 (0.579-20.485) | | | | 0.742 (0.534-14.447) | 0.149 |
| VB1 | 50--150nmolL | 47.307 (33.902-63.790) | | | | 48.884 (34.516-62.727) | 0.959 |
| VB12 | 200--900pg/L | 262.618 (66.786-356.621) | | | | 269.573 (84.550-359.680) | 0.807 |
| VB2 | >200ug/L | 249.850 (92.496-372.892) | | | | 253.113 (164.956-341.044) | 0.519 |
| VB6 | 14.6--72.9umol/L | 27.648 (18.595-38.373) | | | | 30.457 (22.401-38.931) | 0.091 |
| VB9 | 6.8--36.3nmol/L | 11.079 (8.435-17.492) | | | | 11.475 (8.970-17.844) | 0.398 |
| VC | 34--114umol/L | 34.680 (30.126-39.293) | | | | 34.303 (30.420-39.785) | 0.638 |
| VE | 10--15ug/mL | 11.125 (10.794-12.223) | | | | 11.141 (10.734-11.933) | 0.780 |

25(OH)D, 25-hydroxyvitamin D; VA, vitamin A; VB1, vitamin B1; VB2, vitamin B2; VB6, vitamin B6; VB9, vitamin B9; VB12, vitamin B12; VC, vitamin C; VE, vitamin E; ADHD, attention-deficit/hyperactivity disorder. *P* <0.05 was considered statistically significant.

**Table S4. Laboratory characteristics of children in cases and healthy controls**

|  |  | | | Median (IQR) | | |  |
| --- | --- | --- | --- | --- | --- | --- | --- |
|  | Normal Range | | | Cases (Growth disorders) | | Healthy | *P* |
| **Growth retardation** | |  | |  | |  |  |
| 25(OH)D | 75--250nmol/L | | | 72.341 (54.219-96.549) | | 75.330 (56.890-101.085) | **<0.001** |
| VA | 0.52--2.2umol/L | | | 0.775 (0.551-25.465) | | 0.742 (0.534-14.447) | **<0.001** |
| VB1 | 50--150nmolL | | | 46.195 (31.844-60.335) | | 48.884 (34.516-62.727) | **<0.001** |
| VB12 | 200--900pg/L | | | 262.166 (32.390-359.537) | | 269.573 (84.550-359.680) | **<0.001** |
| VB2 | >200ug/L | | | 238.986 (54.606-330.762) | | 253.113 (164.956-341.044) | **<0.001** |
| VB6 | 14.6--72.9umol/L | | | 30.624 (22.844-38.897) | | 30.457 (22.401-38.931) | 0.232 |
| VB9 | 6.8--36.3nmol/L | | | 11.345 (8.836-30.395) | | 11.475 (8.970-17.844) | 0.625 |
| VC | 34--114umol/L | | | 33.817 (29.524-39.070) | | 34.303 (30.420-39.785) | **0.001** |
| VE | 10--15ug/mL | | | 11.212 (10.810-12.243) | | 11.141 (10.734-11.933) | **<0.001** |
| **Obesity** |  | | |  | |  |  |
| 25(OH)D | 75--250nmol/L | | | 57.661 (46.385-75.520) | | 75.330 (56.890-101.085) | **<0.001** |
| VA | 0.52--2.2umol/L | | | 0.870 (0.625-19.636) | | 0.742 (0.534-14.447) | **<0.001** |
| VB1 | 50--150nmolL | | | 48.971 (34.201-62.411) | | 48.884 (34.516-62.727) | 0.710 |
| VB12 | 200--900pg/L | | | 261.635 (40.947-341.583) | | 269.573 (84.550-359.680) | 0.262 |
| VB2 | >200ug/L | | | 261.924 (76.735-367.800) | | 253.113 (164.956-341.044) | 0.232 |
| VB6 | 14.6--72.9umol/L | | | 30.941 (22.897-39.952) | | 30.457 (22.401-38.931) | 0.214 |
| VB9 | 6.8--36.3nmol/L | | | 11.644 (8.707-22.220) | | 11.475 (8.970-17.844) | 0.882 |
| VC | 34--114umol/L | | | 35.917 (31.775-42.799) | | 34.303 (30.420-39.785) | **<0.001** |
| VE | 10--15ug/mL | | | 11.246 (10.797-12.348) | | 11.141 (10.734-11.933) | 0.054 |
| **Malnutrition** |  | | |  | |  |  |
| 25(OH)D | 75--250nmol/L | | | 77.729 (56.572-118.298) | | 75.330 (56.890-101.085) | 0.130 |
| VA | 0.52--2.2umol/L | | | 0.851 (0.579-39.541) | | 0.742 (0.534-14.447) | **0.001** |
| VB1 | 50--150nmolL | | | 46.633 (28.936-61.639) | | 48.884 (34.516-62.727) | **0.007** |
| VB12 | 200--900pg/L | | | 239.546 (10.694-330.763) | | 269.573 (84.550-359.680) | **<0.001** |
| VB2 | >200ug/L | | | 221.408 (38.423-325.529) | | 253.113 (164.956-341.044) | **<0.001** |
| VB6 | 14.6--72.9umol/L | | | 31.345 (23.064-38.489) | | 30.457 (22.401-38.931) | 0.324 |
| VB9 | 6.8--36.3nmol/L | | | 12.504 (8.850-34.750) | | 11.475 (8.970-17.844) | **0.018** |
| VC | 34--114umol/L | | | 33.708 (28.652-41.744) | | 34.303 (30.420-39.785) | 0.179 |
| VE | 10--15ug/mL | | | 11.230 (10.775-12.455) | | 11.141 (10.734-11.933) | 0.071 |
| **Breast development** | | |  | |  |  |  |
| 25(OH)D | 75--250nmol/L | | | 52.556 (39.718-68.698) | | 75.330 (56.890-101.085) | **<0.001** |
| VA | 0.52--2.2umol/L | | | 0.762 (0.574-14.811) | | 0.742 (0.534-14.447) | 0.609 |
| VB1 | 50--150nmolL | | | 49.089 (35.209-61.980) | | 48.884 (34.516-62.727) | 0.672 |
| VB12 | 200--900pg/L | | | 285.932 (178.243-360.589) | | 269.573 (84.550-359.680) | 0.387 |
| VB2 | >200ug/L | | | 265.208 (191.162-368.425) | | 253.113 (164.956-341.044) | 0.355 |
| VB6 | 14.6--72.9umol/L | | | 32.497 (22.198-39.067) | | 30.457 (22.401-38.931) | 0.601 |
| VB9 | 6.8--36.3nmol/L | | | 11.045 (8.920-14.813) | | 11.475 (8.970-17.844) | 0.176 |
| VC | 34--114umol/L | | | 35.322 (31.937-40.761) | | 34.303 (30.420-39.785) | 0.070 |
| VE | 10--15ug/mL | | | 11.163 (10.778-11.989) | | 11.141 (10.734-11.933) | 0.858 |

25(OH)D, 25-hydroxyvitamin D; VA, vitamin A; VB1, vitamin B1; VB2, vitamin B2; VB6, vitamin B6; VB9, vitamin B9; VB12, vitamin B12; VC, vitamin C; VE, vitamin E; *P* <0.05 was considered statistically significant.
